# The variable morphology of suprascapular nerve and vessels at suprascapular notch: a proposal for classification and its potential clinical implications

**DOI:** 10.1007/s00167-014-2937-1

**Published:** 2014-03-18

**Authors:** Michał Polguj, Jacek Rożniecki, Marcin Sibiński, Andrzej Grzegorzewski, Agata Majos, Mirosław Topol

**Affiliations:** 1 Department of Angiology, Medical University of Łódź, Narutowicza 60, 90-136 Łódź, Poland; 2 Department of Neurology, Medical University of Łódź, Łódź, Poland; 3 Clinic of Orthopedic and Pediatric Orthopedics, Medical University of Łódź, Łódź, Poland; 4 Department of Radiology, Medical University of Łódź, Łódź, Poland; 5 Department of Normal and Clinical Anatomy, Medical University of Łódź, Łódź, Poland

**Keywords:** Suprascapular nerve entrapment, Anatomical variation, Suprascapular nerve, Suprascapular artery, Suprascapular vein

## Abstract

**Purpose:**

The most common place for suprascapular nerve entrapment is the suprascapular notch. The aim of the study was to determine the morphological variation of the location of the suprascapular nerve, artery and vein, and measure the reduction in size of the suprascapular opening in each type of the passage.

**Methods:**

A total of 106 human formalin-fixed cadaveric shoulders were included in the study. After dissection of the suprascapular region, the topography of the suprascapular nerve, artery and vein was evaluated. Additionally, the area of the suprascapular opening was measured using professional image analysis software.

**Results:**

Four arrangements of the suprascapular vein, artery and nerve were distinguished with regard to the superior transverse scapular ligament: type I (61.3 %) (suprascapular artery was running above ligament, while suprascapular vein and nerve below it), type II (17 %) (both vessels pass above ligament, while nerve passes under it), type III (12.3 %) (suprascapular vessels and nerve lie under ligament) and type IV (9.4 %), which comprises the other variants of these structures. Statistically significant differences regarding the suprascapular opening were observed between the specimens with types II and III. Anterior coracoscapular ligaments were present in 55 from 106 shoulders.

**Conclusion:**

The morphological variations described in this study are necessary to better understand the possible anatomical conditions which may promote suprascapular nerve entrapment (especially type III). They may be useful during open and endoscopic procedures at the suprascapular notch to prevent such complications as unexpected bleeding.

## Introduction

The natural passage of the suprascapular nerve provides several locations where it is predisposed to compression and injury. According to orthopaedic surgeons, the most common point for nerve compression and traction injury is the suprascapular notch [16, 26]. The suprascapular nerve is the structure that always passes under the superior transverse scapular ligament [20, 21, 24]. However, the location of the associated suprascapular artery and vein is highly variable [22, 23, 25].

In the etiopathology of suprascapular nerve entrapment, less attention has been paid to the topography of the complete suprascapular triad: the nerve, artery and vein. Nevertheless, this is a very important subject, because the route taken by the suprascapular artery under the superior transverse scapular ligament could be one of the possible causes of suprascapular nerve entrapment [2, 22, 23].

This article offers a new classification of the suprascapular nerve, artery and vein topography at the suprascapular notch region. Our study uses image analysis software to present a precise description of the space below the superior transverse scapular ligament for the passage of the suprascapular nerve. Furthermore, earlier observations of the suprascapular triad have been only macroscopic and approximate, and not supported by any calculations, and the present study is the first to take this approach. Additionally, the present study analyses the topography of the anterior coracoscapular ligament upon entry to the suprascapular nerve passage.

## Materials and methods

The study was performed on 106 formalin-fixed cadaveric shoulders (55 right and 51 left) collected in the Department of Anatomy, Medical University of Łódź. In all cadaveric shoulders, after horizontal incision along the clavicle, the skin was separated from the trapezius, deltoid and pectoralis major muscles. Next, the trapezius and the deltoid muscles were disinserted and reclined to expose the supra- and infraspinatus muscles. After retraction of the supraspinatus muscle from the supraspinatus fossa, the superior border of the scapula and superior transverse scapular ligament was visualized. The morphological characteristics of the structures in the suprascapular notch region, the relationship of the suprascapular nerve, artery and vein with the other structures and the presentation of any abnormal masses in this area were noted.

**Fig. 1:**
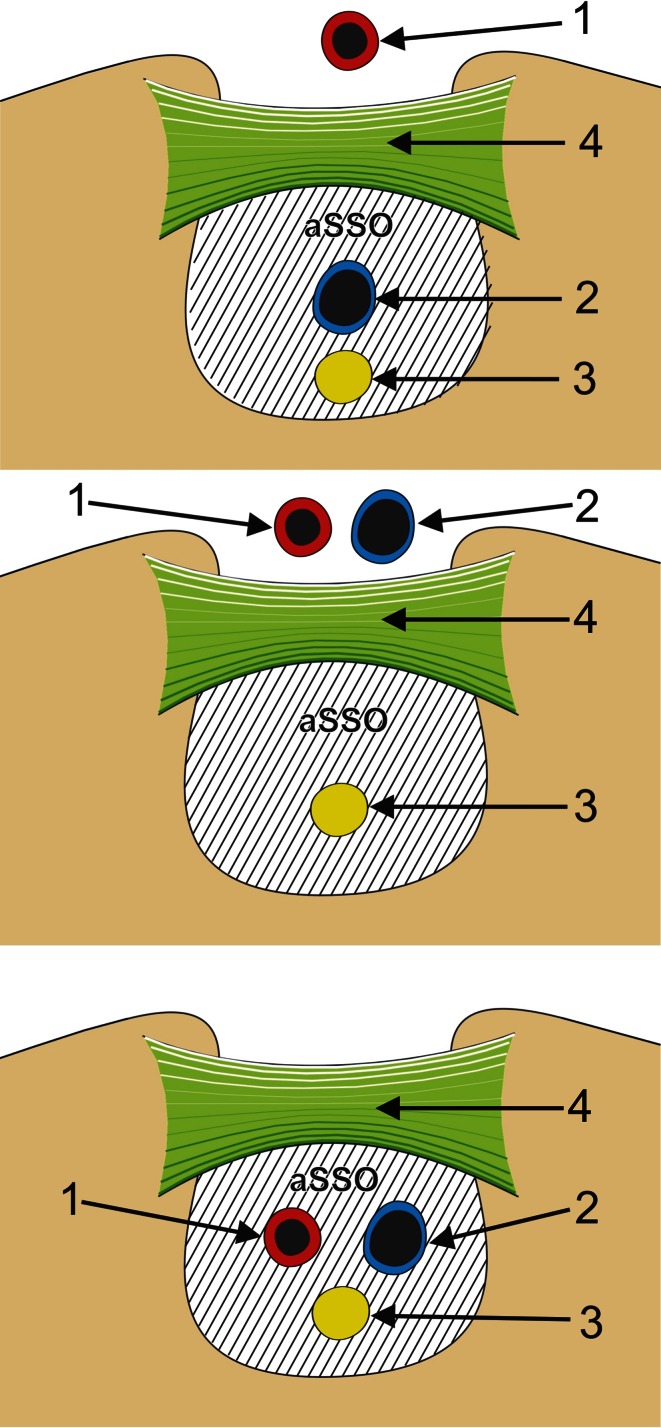
Schematic arrangement of measurements of the suprascapular opening in different types of suprascapular nerve, artery and vein arrangement. *1* suprascapular artery, *2* suprascapular vein, *3* suprascapular nerve, *4* superior transverse scapular ligament, *aSSO* area of the suprascapular opening

To normalize measurements, all photographic documentation was obtained from a standardized position of the camera and shoulder. The same scale was used for all measurements. The shoulders were fixed with an adjustable clamp and ring stand at the same distance from the camera for each observation. Digital photographic documentation was processed in MultiScanBase v.18.03 software (Computer Scanning System II, Warsaw, Poland) to obtain the area of the suprascapular opening (aSSO) in various location of the suprascapular triad (Fig. 1). Each measurement was taken three times by one investigator, and the mean of the values was calculated. For the sake of the investigation, the area of the suprascapular opening was defined as the area limited superiorly by the inferior border of the superior transverse scapular ligament, laterally and medially by the osseous walls of the suprascapular notch, and inferiorly by the superior border of the anterior coracoscapular ligament or inferior border of the suprascapular notch for specimens without an anterior coracoscapular ligament. The diameters of the suprascapular nerve and artery were also measured.

The sex and age of the donors were unknown. The research project was approved by the Bioethics Commission of the Medical University of Łódź (protocol ID no. RNN/12/10/KE).

### Statistical analysis

All data analysis was performed using Statistica 10 software (StatSoft Polska, Cracow, Poland). The Shapiro–Wilk test was used to confirm whether the distribution of continuous variables was normal. The Mann–Whitney test was used to assess the statistical differences regarding the area of the suprascapular opening between samples with an anterior coracoscapular ligament and those without, for each type of suprascapular triad topography. To evaluate the intra-observer and inter-observer repeatability of measurements of the area of the suprascapular opening, this parameter was calculated in 20 specimens again by the same researcher and by the second author. Linear regression analysis was used to calculate the *R* ^2^ value, which determined the level of agreement. The bias was assessed with the Bland–Altman plot, which depicts the percentile difference between two measurements (Y axis) against their mean (X axis). Mean, standard deviation, median, minimum and maximum for continuous variables were used as descriptive statistics. A *p* level of <0.05 was accepted as statistically significant.

## Results

In all the investigated cadaveric shoulders, a single suprascapular nerve was found. It was found to pass below the superior transverse scapular ligament near the inferior wall of the suprascapular notch or the superior border of the anterior coracoscapular ligament.

**Fig. 2:**
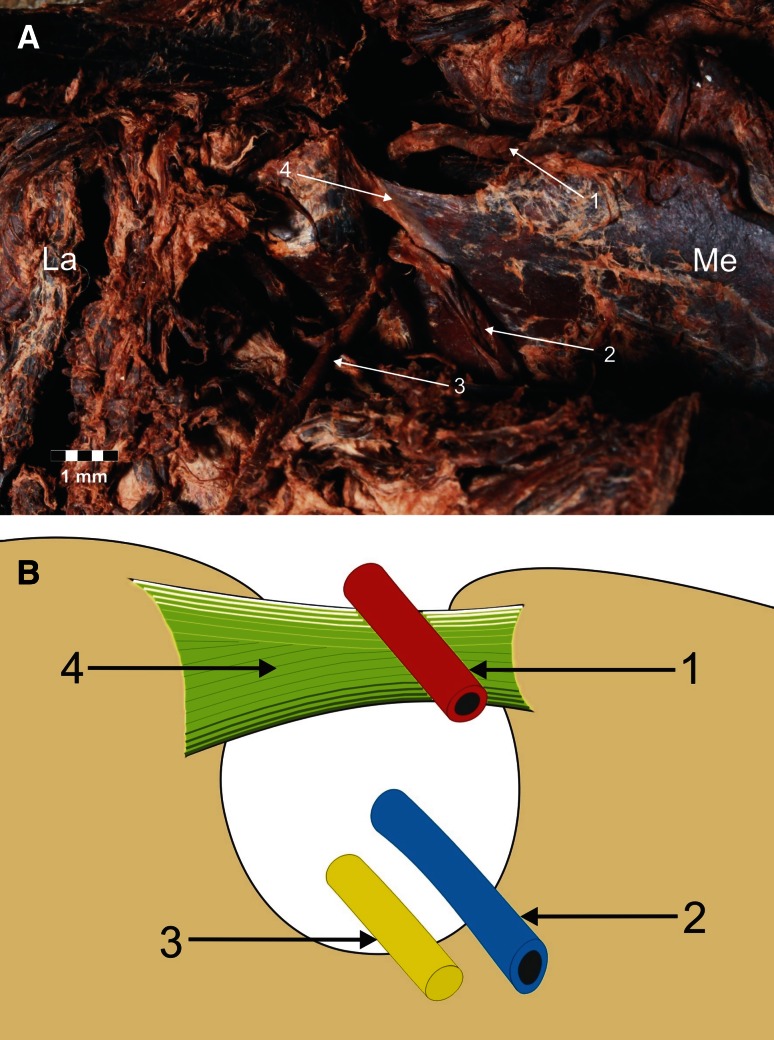
Type I arrangement of the suprascapular nerve, artery and vein at the suprascapular notch. **a** Structures at the cadaver, **b** schematic arrangements. *1* suprascapular artery, *2* suprascapular vein, *3* suprascapular nerve, *4* superior transverse scapular ligament. *La* lateral, *Me* medial

**Fig. 3:**
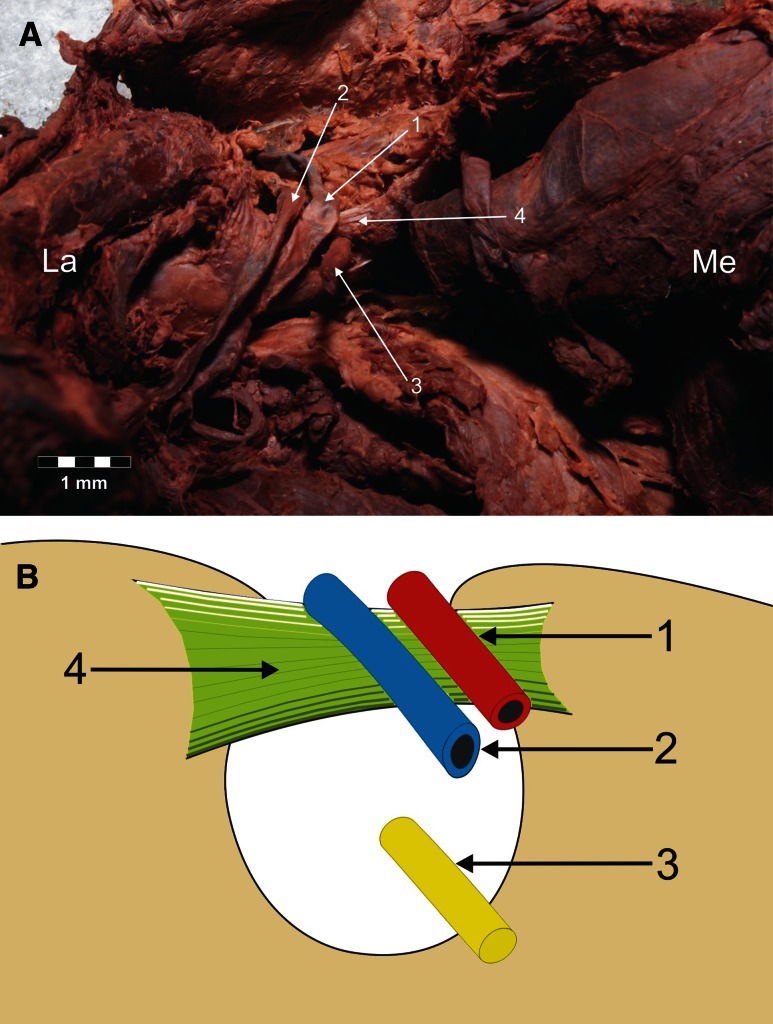
Type II arrangement of the suprascapular nerve, artery and vein location at the suprascapular notch. **a** Structures at the cadaver, **b** schematic arrangements. *1* suprascapular artery, *2* suprascapular vein, *3* suprascapular nerve, *4* superior transverse scapular ligament. *La* lateral, *Me* medial

**Fig. 4:**
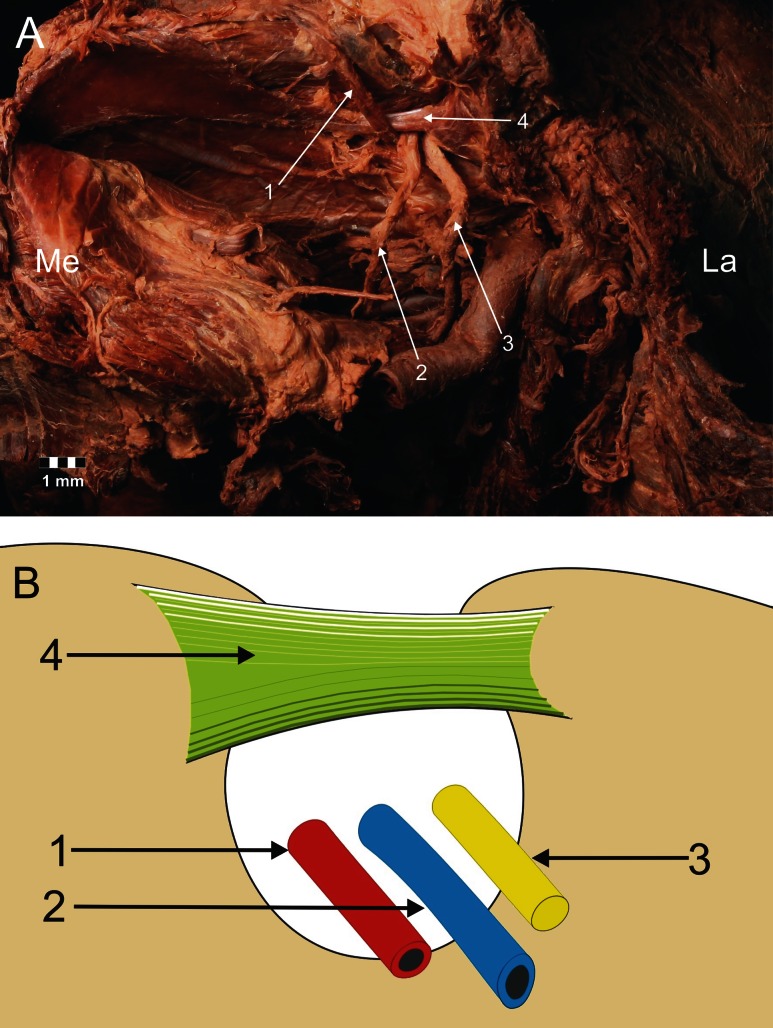
Type III arrangement of the suprascapular nerve, artery and vein location at the suprascapular notch. **a** Structures at the cadaver, **b** schematic arrangements. *1* suprascapular artery, *2* suprascapular vein, *3* suprascapular nerve, *4* superior transverse scapular ligament. *La* lateral, *Me* medial

**Fig. 5:**
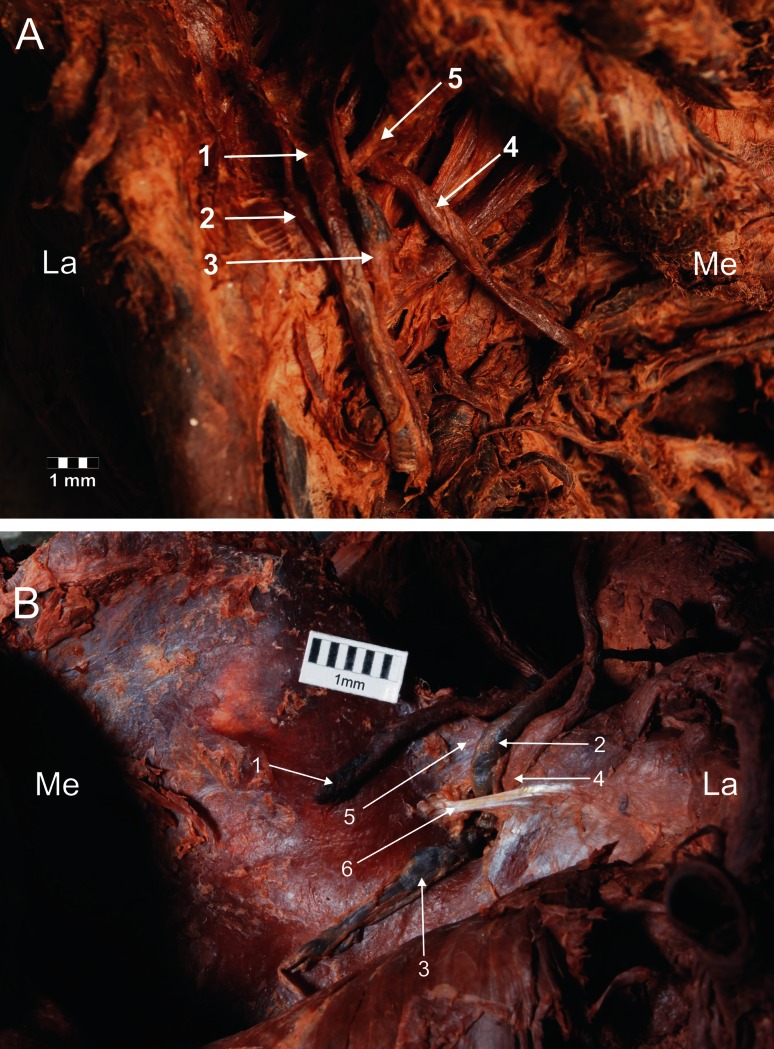
Type IV arrangement of the suprascapular nerve, artery and vein location at the suprascapular notch (double suprascapular vein). *1* suprascapular artery, *2*, *3* suprascapular veins, *4* suprascapular nerve, *5* superior transverse scapular ligament, *6* anterior coracoscapular ligament. *La* lateral, *Me* medial

Four types of suprascapular vein, artery and nerve location were distinguished in relation to the superior transverse scapular ligament. In type I (65/106–61.3 %), the suprascapular artery runs above the superior transverse scapular ligament, while the suprascapular vein and nerve run below it (Fig. 2). Type II (18/106–17 %) included specimens in which two vessels pass above the superior transverse scapular ligament, while the nerve is situated beneath it (Fig. 3). In type III (13/106–12.3 %), the suprascapular vessels and nerve lay directly under the ligament (Fig. 4). Type IV (10/106–9.4 %) comprised the other variants of these structures: among others, the occurrence of the accessory suprascapular veins, and the cases in which the analysed structures pass under the anterior coracoscapular ligament (Fig. 5). Double suprascapular veins were found on two extremities (2.4 %) (Fig. 5): in the first case, it passed above the superior transverse scapular ligament (Fig. 5a), and in the second case, below the anterior coracoscapular ligament (Fig. 5b).

The mean surface areas of the suprascapular opening in the specimens with types I, II, III and IV of the suprascapular triad location were 30.9 ± 18.4, 32.1 ± 14, 22.6 ± 12.4 and 30.7 ± 20.9 mm^2^, respectively (Table 1). According to the Mann–Whitney test (*p* > 0.05), only the mean surface areas of the suprascapular openings in types II and III specimens were significantly different (*p* = 0.0263).

**Table 1:** Area of the suprascapular opening in different types of suprascapular vein, artery and nerve location at the suprascapular notch

Measurement	Type	Number of specimens (*n*)	Mean	SD	Min–max	Median
Area of the suprascapular opening (mm^2^)	Type I	65	30.9	18.4	4.6–85.4	25.5
	Type II	18	32.1	14	6.3–68.2	29.7
	Type III	13	22.6	12.4	10.2–58.3	18.3
	Type IV	10	30.7	20.9	10.7–76.8	23.5

*SD* standard deviation, *Min* minimum, *Max* maximum

**Fig. 6:**
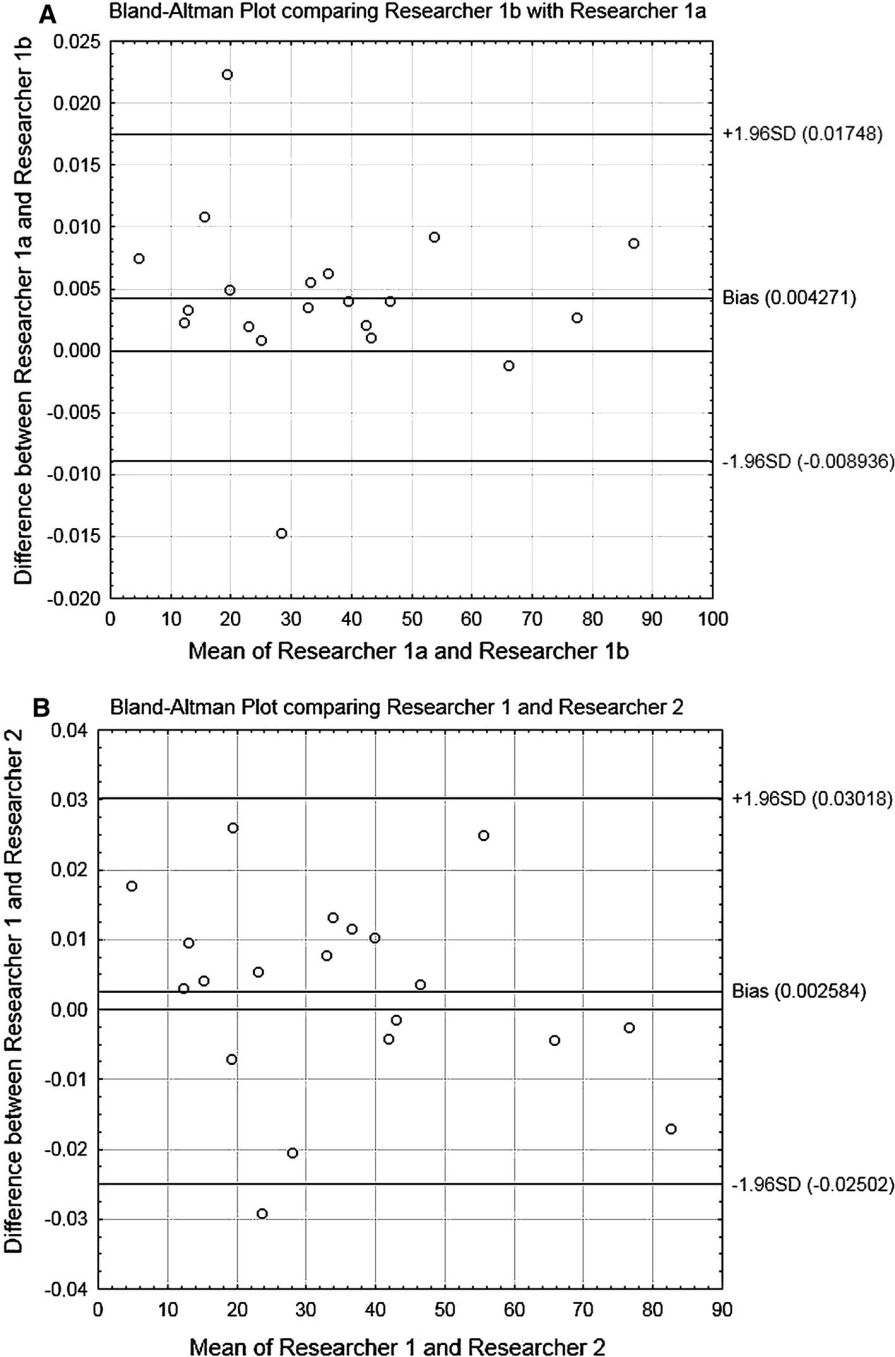
A Bland–Altman plot depicting the bias and limits of measurements of the area of the suprascapular opening (**a**) one researcher (two measurements by the same researcher—1a and 1b)—intra-observer repeatability; (**b**) two researchers (1 and 2)—inter-observer repeatability

An anterior coracoscapular ligament was found in 55 of the 106 shoulders (51.9 %) (Figs. 5b, 6). Taking into consideration the types of the suprascapular vein, artery and nerve location at the suprascapular notch, an anterior coracoscapular ligament was found in 19 type II specimens, 15 type III specimens, 12 type I specimens and 9 type IV specimens. The suprascapular nerve passed under the anterior coracoscapular ligament in three of the 106 specimens (2.8 %) and superior to it in 52 (49.1 %). The mean diameters of the suprascapular nerve and artery were 2.48 ± 0.6 mm and 2.33 ± 0.5 mm, respectively (mean and standard deviation).

The intra-observer and inter-observer degrees of agreement were *R* ^2^ = 0.9985 and *R* ^2^ = 0.9895, respectively. Details concerning limits of agreement and bias are presented in Bland–Altman plots (Fig. 6). The Bland–Altman plot and *R* ^2^ value confirm that digital method of measurement is a very reliable technique throughout the range of area of the suprascapular opening.

## Discussion

The most important finding of the present study was the quantitative analysis of the space available for the path of the suprascapular nerve in different locations within the suprascapular triad at the suprascapular notch. According to our study, specimens with a type III suprascapular triad arrangement, i.e. the suprascapular vessels and nerve lie beneath the superior transverse scapular ligament, were found to have the smallest mean area of the suprascapular opening compared to the others. Such observation is important for a better understanding of the possible morphological conditions that may promote suprascapular nerve entrapment.

The results of this paper may also have clinical value in endoscopic and open procedures of suprascapular nerve decompression. The topography of the suprascapular triad should be taken into consideration as an important risk factor in the formation of suprascapular nerve entrapment, particularly the case of the type III arrangement. Such a condition could be further exacerbated by the presence of any concomitant, extraordinary mass, such as a paralabral ganglion cyst. Although this may be a sufficient, and probably the most common, cause of suprascapular nerve neuropathy, hematoma or any neoplastic tumour (i.e. sarcoma) could also have an influence [5].

One classification of the configuration of the suprascapular vein, artery and nerve at the suprascapular notch has previously been described [25]. Our classification is partially similar to Yang’s [25]. The most common arrangement, according to our classification, is the type I suprascapular triad topography and was presented in 65 shoulders (61.3 %). Although this arrangement is comparable with Yang’s type IIC, the rates of occurrence are significantly different, with only three shoulders being graded as IIC according to Yang et al. [25]. Also, although the configurations of the suprascapular triad according to Yang’s type I and our type II are similar, the frequency of occurrence was found to be considerably lower (17 %) in our study than that of Yang et al. [25] (59.4 %). Type III is the same according to both classifications, and its frequency in our study (12.3 %) was found to be similar to Yang et al. [25] (10.9 %). It may be that such significant differences in frequency of location of the suprascapular nerve and vessels at the suprascapular notch depend on the studied population. It would be reasonable to assume that its occurrence varies worldwide based on population; indeed, the incidence of the anterior coracoscapular ligament has been found to vary from 18.8 to 60.0 % depending on population [1, 2, 18]. In the present study, the frequency of occurrence of the anterior coracoscapular ligament was 51.9 %, which was lower than that described by Avery et al. [1] (60 %) in the American population. However, it was higher than seen by Bayramoglu et al. [2] (18.8 %) in the Turkish population, and Piyawinijwong and Tantipoon [18] (28 %) in the Thai population.

The problem of suprascapular nerve entrapment formation is complex and depends also on factors such as the shape of the suprascapular notch [14, 21], the shape of the superior transverse scapular ligament [19, 20], the presence of hypertrophied suprascapular muscle [7], a completely ossified superior transverse scapular ligament [8, 13] or the presence of the a spinoglenoidal ligament [17]. However, the most important factor in suprascapular nerve entrapment formation is probably the position of the suprascapular nerve [15, 22]. Our description of the suprascapular nerve course in the suprascapular notch is similar to those of Bayramoglu et al. [2], Greiner et al. [10] and Piyawinijwong and Tantipoon [18]. In their studies, the suprascapular nerve was always seen to pass between the superior transverse scapular ligament and anterior coracoscapular ligament. In our study, it was found to run superior to the anterior coracoscapular ligament in 52 of 55 cases and inferior to it in 3 of 55. The diameter of the suprascapular nerve measured in the present study is 2.48 ± 0.6 mm (mean ± SD). This value is similar to previous descriptions: 2 mm [11], 3 mm [12], 3.3 mm [1].

Yang et al. [25] noted the presence of a single suprascapular artery in all shoulders, and double suprascapular veins in 19.4 % of them. These findings were significantly higher than those of our study: accessory suprascapular veins were only observed in 2.4 % of the cases examined in the present study. Chen and adds [6] reported an accessory suprascapular artery which accompanied the suprascapular nerve under the superior transverse scapular ligament. Although no such vessels were found in the present study, this is an important finding because they probably reduce space for suprascapular nerve passage and also contribute to the supply of the middle third of the clavicle by several periosteal branches.

The nonspecific symptoms of suprascapular nerve neuropathy can result in late diagnosis when atrophy of the supraspinatus and infraspinatus muscles is present. As this mainly occurs in patients under 38 years of age, it would seem to be a demographic problem [9, 26]. According to Gosk et al. [9], the outcome of surgery depends on the length of time between symptom onset and the surgery itself, and the pathology underlying the nerve compression. Therefore, studies on anatomical variations of structures located at the suprascapular region should be helpful in the diagnosis and treatment of this pathology.

Our study had some limitations. In the proposed classification, type IV is a heterogeneous group that comprises cases with suprascapular openings displaying a wide range of areas. Thus, without knowing the morphology of particular type IV suprascapular notch, no information about structures occupying the suprascapular notch can be obtained. Moreover, as the dissected shoulders were obtained from different donors, it was not possible to evaluate the symmetry of the triad types. Finally, as the sex of specimens remained unknown, no conclusions could be reached regarding inter-gender differences in triad types. Nevertheless, our study is the first to use a quantitative method to discern the influence of suprascapular triad topography on the space available for the suprascapular nerve travelling under the superior transverse scapular ligament. This is significant, because according to our results, the type III arrangement allows less space for the passage of the suprascapular nerve. Knowledge of the topography of the suprascapular triad may also prevent unforeseen bleeding after suprascapular artery or vein injury [3, 4, 16].

## Conclusion

The new classification of the suprascapular triad topography presented herein and the quantitative analysis of the space available for the path of the suprascapular nerve are important for a better understanding of the possible morphological conditions that may promote suprascapular nerve entrapment, especially those with a type III arrangement.
